# Phenotype–Genotype Analysis of Chinese Patients with Early-Onset *LMNA*-Related Muscular Dystrophy

**DOI:** 10.1371/journal.pone.0129699

**Published:** 2015-06-22

**Authors:** Dandan Tan, Haipo Yang, Yun Yuan, Carsten Bonnemann, Xingzhi Chang, Shuang Wang, Yuchen Wu, Xiru Wu, Hui Xiong

**Affiliations:** 1 Department of Pediatric, Peking University First Hospital, Beijing, China; 2 Department of Neurology, The First Affiliated Hospital of Nanchang University, Nanchang, China; 3 Department of Neurology, Peking University First Hospital, Beijing, China; 4 Neuromuscular and Neurogenetic Disorders of Childhood Section, National Institute of Neurological Disorders and Stroke/NIH, Bethesda, MD, United States of America; Second Affiliated Hospital, Zhejiang University, CHINA

## Abstract

This study aimed to analyze the correlation between the phenotype and genotype of Chinese patients with early-onset lamin A (LMNA)-related muscular dystrophy (MD). The clinical and myopathological data of 21 Chinese pediatric patients with early-onset *LMNA*-related MD were collected and analyzed. *LMNA* gene mutation analysis was performed by direct sequencing of genomic DNA. Sublocalization of wild-type and mutant proteins were observed by immunofluorescence using cultured fibroblasts and human embryonic kidney 293 (HEK 293) cell. Seven patients were diagnosed with Emery-Dreifuss muscular dystrophy (EDMD) and 14 were diagnosed with *LMNA*-associated congenital muscular dystrophy (L-CMD). Four biopsy specimens from the L-CMD cases exhibited inflammatory changes. Abnormal nuclear morphology was observed with both transmission electron microscopy and lamin A/C staining. We identified 10 novel and nine known *LMNA* gene mutations in the 21 patients. Some mutations (c.91G>A, c.94_96delAAG, c.116A>G, c.745C>T, c.746G>A, and c.1580G>C) were well correlated with EDMD or L-CMD. *LMNA*-related MD has a common symptom triad of muscle weakness, joint contractures, and cardiac involvement, but the severity of symptoms and disease progression differ greatly. Inflammatory change in biopsied muscle is a characteristic of early-stage L-CMD. Phenotype–genotype analysis determines that some mutations are well correlated with *LMNA*-related MD.

## Introduction

The lamin A (*LMNA*) gene (OMIM 150330) is located on chromosome 1q21.1–21.3, encompassing 12 exons and encoding A-type lamins that are nuclear envelope intermediate filament proteins on the inner nuclear membrane. Lamins A/C are the major splice products of A-type lamins, providing a nuclear scaffold for protein complexes, maintaining nuclear structure, regulating gene expression, and playing roles in signaling pathways [1–3]. Mutations in the *LMNA* gene cause a series of rare and diverse diseases called laminopathies [4]. Among them, autosomal Emery–Dreifuss muscular dystrophy (EDMD, OMIM 181350), *LMNA*-associated congenital muscular dystrophy (L-CMD, OMIM 613205), limb-girdle muscular dystrophy type 1B (LGMD1B, OMIM 159001) are classified as *LMNA*-related muscular dystrophies. Although they exhibit common features of muscular dystrophy, their presentation and clinical severity can differ greatly.

The *LMNA*-related muscular dystrophy with the earliest onset is L-CMD, whose symptoms manifest in the first few months of life or even during the fetal period. Patients with L-CMD can have decreased fetal or newborn movements and significant early delay and/or decline of motor development [5]. They may initially present with proximal limb and cervical-axial muscle weakness, and generalized hypotonia. Strikingly, some present with poor head control, including “dropped head syndrome”, due to neck muscle weakness [6]. The progression of L-CMD is the fastest of the *LMNA*-related muscular dystrophies, and its clinical manifestation is the most serious. Most patients are unable to walk or sit unaided in childhood, and some even require early respiratory or nutritional support, or present with early cardiac disease. EDMD mostly presents in early childhood with a myopathic gait caused by weakness, spinal deformity, and ankle contractures. The clinical triad of EDMD is early contracture of the elbows, Achilles tendons, and neck extensors; slowly progressive muscle wasting and scapuloperoneal weakness; and abnormality of cardiac conduction [7]. By comparison, limb-girdle muscle weakness in patients with LGMD1B begins in childhood, continuing to adulthood. Elbow contracture and restrictive neck flexion are not evident at the early stage; the muscle weakness of LGMD1B is stable, and most patients are able to walk unaided.

While the suggestive clinical features of the disease contribute to the diagnosis, the exact correlation between phenotype and genotype in *LMNA*-related muscular dystrophy remains unclear, as does the mechanism of specific skeletal muscle involvement in laminopathies. In our study, we analyzed the clinical data and *LMNA* gene mutations in 21 patients to identify the phenotypic spectrum in the Chinese population and establish correlations between the phenotypes and genotypes.

## Patients and Methods

### Ethics statement

The study was approved by the Ethics Committee of the Peking University First Hospital. Informed written consent was obtained to participate in the study, as well as publish medical data from controls, in addition to the parents or guardians of the children.

### Subjects

Twenty-nine pediatric patients from Peking University First Hospital between 2008 and 2013 were clinically diagnosed *LMNA*- related muscular dystrophies but 21 were made a genetically definite diagnosis. These 21 cases were recruited retrospectively. The following clinical variables were recorded (Table 1): age, sex, initial symptoms, motor function, contracture, deformed spine, and cardiac involvement. The routine laboratory tests included an electrocardiogram, electromyogram, serum creatine kinase (CK) level, and cerebral magnetic resonance imaging (MRI).

**Table 1:** Clinical and neuroradiological findings of the patients.

**Patient No./ sex/age at last review**	Presenting Features (age)	Motor function/muscle weakness at last review	Contractures	Deep tendon reflex	Deformed spine	CK (IU/L)	ECG	EMG	Muscle biopsy(age)	Brain MRI
1/M/6y	Proximal weakness, dropped head (4m)	Never walk; hypotonia; UL: proximal, distal; LL: proximal, distal	Elbow,knee, ankle, hip	Not educed		1248	ND	Neurogen-ic injury	ND	Normal
2/F/10y	Myopathic gait(1y)	Nonambulant at 6y; UL:proximal; LL:proximal		Not educed	Lumbar lordosis	451	ND	Myogenic injury	dystrophic changes(10y)	ND
3/M/5y	Proximal weakness, hold head poorly (13m)	Ambulant; mild hypotonia; UL:proximal; LL:proximal	Knee, ankle	Not educed		749	Sinus arrhythmia	Normal	dystrophic changes(5y)	Normal
4/M/4y	Proximal weakness, myopathic gait (18mo)	Ambulant; hypotonia; UL:proximal; LL:proximal		Not educed	thoracic lordosis	371	Sinus rhythm	Normal	dystrophic changes(4y)	Normal
5/F/9y	Myopathic gait(2y)	Ambulant; UL:proximal; LL:proximal	Elbow, ankle	Not educed	Scoliosis, rigid spine	244	Sinus rhythm	Normal	ND	Normal
6/F/2y	Proximal weakness, dropped head(4m)	Sit alone; cervical-axial; UL and LL		Not educed	Lordosis	906–1062	Sinus tachycardia	Myogenic injury	dystrophic changes(20m)	Normal
7/F/16m	Limb weakness, dropped head(8m)	Never sit at 16m; hypotonia; UL: proximal; LL: distal	ankle	Not educed		405–1817	Sinus tachycardia	ND	dystrophic changes with inflammation(16m)	Normal
8/F/7y	Proximal weakness(2y)	Ambulant; UL:proximal; LL:proximal	Elbow, knee, ankle	Not educed	Lordosis, rigid spine	623	Sinus arrhythmia	ND	ND	ND
9/F/4y	Proximal weakness(6m), dropped head(2y)	Never walk; dropped head; hypotonia; UL:proximal; LL:proximal; Facial	Ankle, hip	Not educed	Lordosis,	882	Sinus rhythm	Myogenic injury	dystrophic changes with inflammation(18m)	Normal
10/M/5y	Proximal weakness(4y)	Ambulant; UL:proximal,; LL:proximal	Ankle	Not educed	Lordosis	554	Sinus rhythm	Myogenic injury	Myopathic Changes (5y)	ND
11/F/9m	Proximal weakness, dropped head(4m)	Never sit at 9m; hypotonia; UL:proximal;LL:proximal	Ankle	Not educed		3766	Sinus arrhythmia	Normal	Inflammatory myopathy (8m)	Normal
12/M/19m	Proximal weakness,hold head poorly(10m)	Nonambulant at 19m; hypotonia; UL:proximal,distal; LL:proximal,distal		Not educed		511	Ventricular tachycardia	Myogenic injury	Muscle dystrophic changes (14m)	Normal
13/M/2y	Truncal hypotonia, dropped head(6m)	Ambulant at 23m		Active		1653	Sinus rhythm	Myogenic injury	Inflammatory myopathy (10m)	Normal
14/M/3y	Proximal weakness(2y)	Ambulant at 3y; mild hypotonia; UL:proximal; LL:proximal		Not educed	Lordosis	1046	Right bundle-branch–block	Myogenic injury	Muscle dystrophic changes (3y)	ND
15/F/4y	myopathic gait(15m)	Ambulant at 3y; mild hypotonia; UL:proximal; LL:proximal		Weake-ned	Lordosis,mild rigid spine	427	Sinus arrhythmia	Myogenic injury	Myopathic changes(3y)	ND
16/M/6y	myopathic gait(5y)	Ambulant at 14m, mild hypotonia; UL:proximal; LL: distal	Elbow, ankle,	Not educed		487~568	Sinus rhythm	Normal	ND	ND
17/F/7y	myopathic gait(3y)	Ambulant at 1y; UL:proximal; LL:proximal		Not educed	Lordosis	992	Sinus rhythm	Myogenic injury	ND	ND
18/F/2y	Proximal weakness, dropped head(1y)	Never walk; hypotonia; UL:proximal; LL: proximal	ankle	Not educed		Normal	ND	Myogenic injury	dystrophic changes(21m)	Normal
19/M/1y	Proximal weakness, dropped head(1y)	Sit alone at 7m; hypotonia; UL:proximal; LL: proximal	ankle	Not educed	Lordosis	515~ 3238	ND	ND	ND	ND
20/M/7m	Proximal weakness, dropped head(7m)	Never sit at 7m; hypotonia; UL:proximal; LL: proximal	ankle	Not educed	Lordosis	1568	ND	ND	ND	Normal
21/M/5y	myopathic gait(3y)	Ambulant at 18m; UL:proximal; LL:proximal		Not educed		1086	ND	Myogenic injury	ND	ND

CK = creatine kinase; ECG = electrocardiogram; EMG = electromyogram; MRI = magnetic resonance imaging; UL = upper limb; LL = lower limb; ND = not done.

### Muscle biopsy

Open muscle biopsy of the gastrocnemius muscle was carried out on 13 patients, and the fresh frozen muscle specimens were fixed in liquid nitrogen. Frozen sections (8-μm) were processed for conventional histochemical staining. Muscle cell ultrastructure was observed under transmission electron microscopy (JEM-1230; JEOL, Tokyo, Japan).

### Mutation screening

Diagnostic testing and the research protocol were reviewed and approved by the Ethics Committee of the Peking University First Hospital (Beijing, China). DNA was extracted from the blood cells of the patients and their parents. The open reading frame and intron/exon boundary of the *LMNA* gene were PCR-amplified using oligonucleotide primers designed by Primer 5.0 (Premier, Canada). To exclude the possibility that the *LMNA* gene mutation represented polymorphisms, identical genomic fragments from 100 healthy controls of Chinese ethnic origin were amplified and examined by direct sequencing of the PCR products for the presence of new mutations and by comparing with the available databases, such as 1000 Genomes (http://www.1000genomes.org).

### RNA extraction and RT-PCR mutation analysis

Total RNA from the peripheral blood leukocytes of patient 13 and his father were purified using TRIzol (Invitrogen, Carlsbad, California, USA). Reverse transcription (RT) was performed using 100 ng total RNA and Moloney murine leukemia virus reverse transcriptase (Invitrogen) according to the manufacturer’s instructions. The resulting complementary DNA (cDNA) was amplified using a primer pair (forward, 5′-GACCTGGAGGACTCACTGGC-3′; reverse, 5′-CGTGGTGGTGATGGAGCAG-3′) designed to reveal the splicing mutation (c.IVS8-7_14del). The PCR conditions were 5 min at 95°C; 30 cycles of 95°C for 30 s, 61°C for 20 s, and 72°C for 50 s; followed by a final extension at 72°C for 5 min.

### Fibroblast culture and immunofluorescence

Dermal fibroblasts of patient 7 were cultured in Dulbecco’s modified Eagle’s medium (DMEM) containing 20% fetal bovine serum (FBS) and 1% penicillin/streptomycin (PS) in 5% CO_2_ at 37°C. Fibroblasts (60% confluent) were fixed with 4% paraformaldehyde and permeabilized with 0.25% Triton X-100. After blocking with 10% goat serum for 40 min at room temperature, cells were incubated with lamin A/C mouse monoclonal antibody (1:200, 100 μL; 4C11, Cell Signaling Technology, Danvers, MA, USA) at 4°C overnight. The cells were washed, incubated with ﬂuorescence-conjugated secondary antibodies for 1 h at room temperature, washed again, and incubated with Hoechst 33342 (1:4000, 1 µg/mL; Molecular Probes, Eugene, OR, USA) for 40 min at room temperature. After the final wash, cells were observed using laser confocal microscopy.

### HEK 293 cell culture, plasmid construction, plasmid transfection

Human embryonic kidney 293 (HEK 293) cells were cultured in DMEM supplemented with 10% FBS and 0.5% PS in 5% CO_2_ at 37°C. Expression vectors were constructed by cloning human *LMNA* into pEGFP-N1. Utilizing standard PCR-based in vitro mutagenesis (Fast Mutagenesis System Kit; TransGen Biotech, Beijing, China), wild-type *LMNA* was altered to express four mutants: c.143G>C (p.R48P), c.745C>T (p.R249W), c.1117A>G (p.I373V), and c.IVS8-7_14del (p.I497_E536del). Each mutant full-length construct was confirmed by direct DNA sequencing. HEK 293 cells were transfected using Lipofectamine Plus (Invitrogen, Carlsbad, California, USA). Transfected cells were grown at 37°C, harvested at 24 h after transfection, and observed under microscopy. Expression of green fluorescent protein (GFP) in cells containing the wild-type and mutant proteins was observed to determine lamin A/C localization.

## Results

### Clinical characteristics of patients

The 21 patients studied comprised 10 boys and 11 girls who were aged between 7 months and 10 years. Patient 1 was Caucasian and the others were Han Chinese. CK levels were mildly to moderately increased from 244 IU/L to 3766 IU/L and did not correlate with the phenotype. The clinical and neuroradiological findings of the patients are listed in Table 1. Two groups could be distinguished according to the phenotype.

### Group I: L-CMD

Patients 1–4, 6–9, 11–13, and 18–20 were aged between 7 months and 10 years old. Most of the 14 children presented motor development retardation in the first 18 months of life; almost all exhibited progressive proximal limb and axial muscle weakness, joint contractures, and spinal deformation. Only patient 9 exhibited mild facial muscle weakness. As newborns, patients 4, 9, 12, 19, and 20 had feeding difficulties, and patients 11–13, 19, and 20 cried weakly. Patients 1, 11, and 13 had recurrent respiratory tract infections when they were 4–12 months old. Patients 1, 6–7, 9, 11–13, and 18–20 had typical dropped head syndrome. Regarding maximum motor ability, patients 7, 9, 11, 18, and 20 could not control their heads or sit unaided; patients 1, 6, 12, and 19 could sit unaided for a moment; patient 2 could walk with crutches; and patients 3, 4, 8, and 13 could walk independently but had difficulty running, jumping, and going up and down stairs. Only patient 12 had cardiac involvement with ventricular tachycardia. Patient 1 died, but the reason for this was unknown.

### Group II: EDMD

Patients 5, 10, 14–17, and 21 were aged between 3 and 9 years. They all presented with slowly progressive muscle weakness with scapuloperoneal distribution and contractures (elbows, ankles, rigid spine), and normal primary motor milestones, acquiring head control at 3 months and sitting unsupported at 6–7 months. They began walking independently at 12–18 months, and then had difficulties running and jumping, going up and down stairs, and standing up from a squat. Spinal involvement was evident in five patients. All but patient 14 had Trendelenburg gait. Patients 14–16 and 21 had mild hypotonia. Their deep tendon reflexes were not educed or reduced. Incomplete right bundle branch block was disclosed only on the electrocardiogram of patient 14. The father of patient 15 exhibited the same symptoms as his daughter: slowly progressive scapuloperoneal muscle weakness, elbow and neck extensor contracture, rigid spine, and sinus bradycardia (36 beats/min). However, the electrocardiogram of patient 15 indicated sinus arrhythmia.

### Myopathological findings

**Fig 1:**
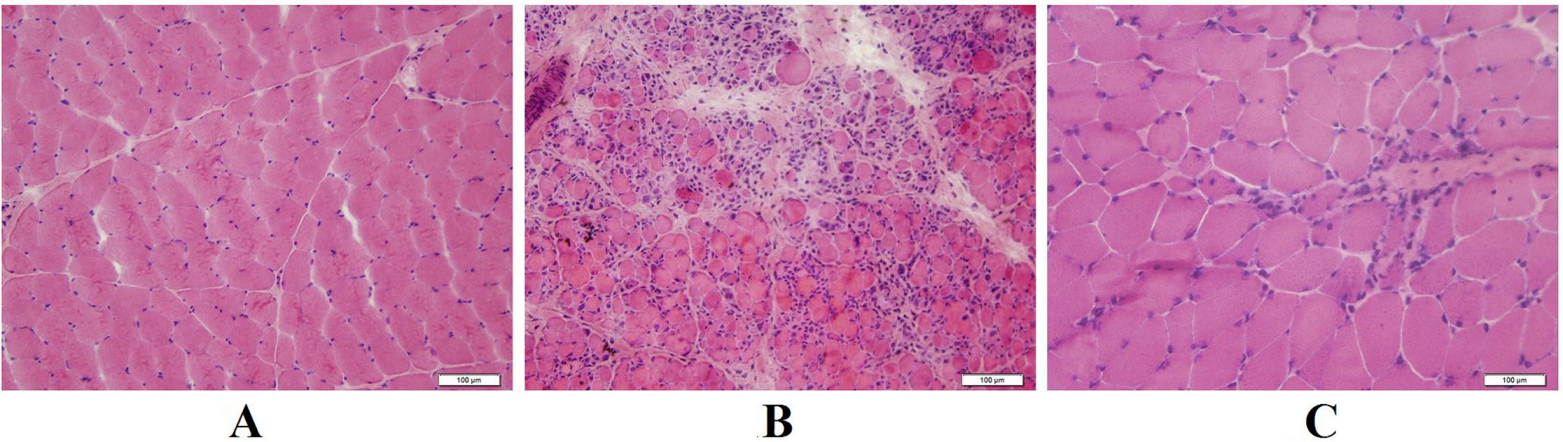
Muscle biopsy specimens stained with hematoxylin and eosin. (A) Normal control (×200). (B) Specimen from patient 11 exhibiting inflammatory cellular infiltration with necrotic and regenerative fibers (×200). (C) Specimen from patient 15 exhibiting mild nuclear transfer and regenerative fibers (×400).

**Fig 2:**
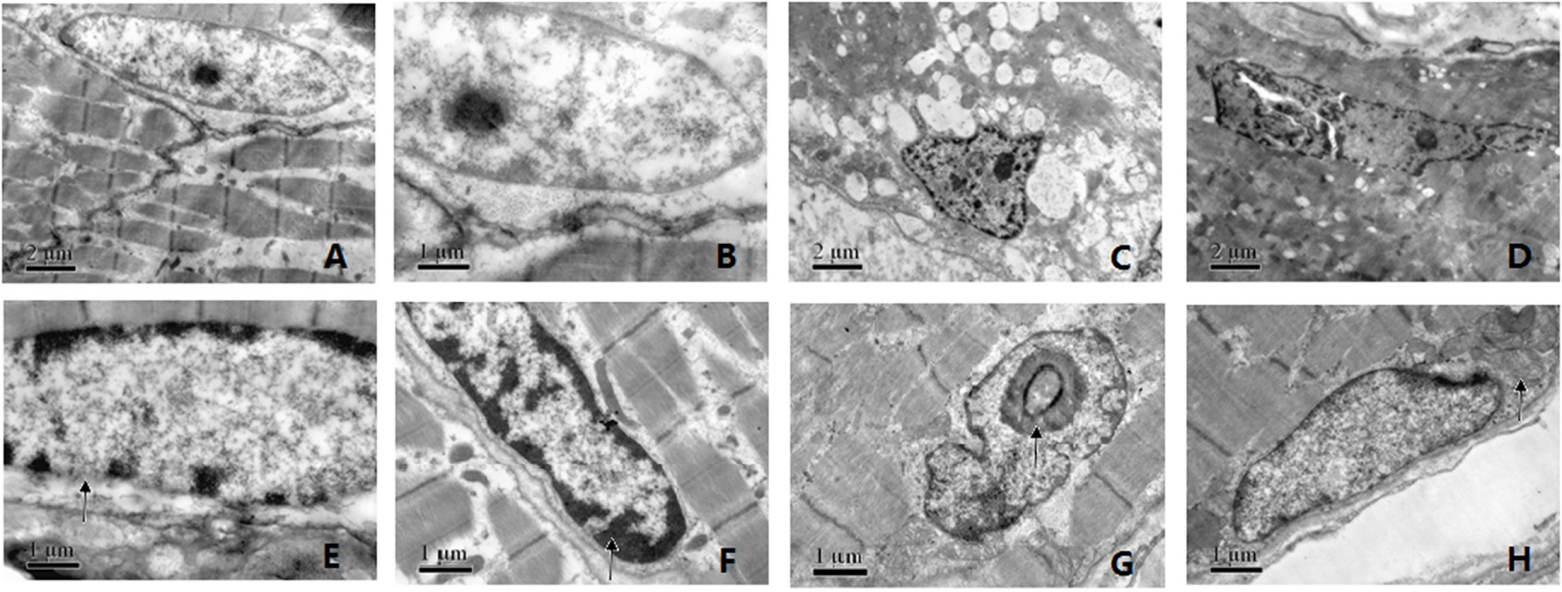
Muscle cell nuclear morphology. A, C, D: ×12000; B, E, F, G, H: ×25000. (A, B) Normal control. (C, D, G) Abnormal nuclear morphology. (C, D, F) Heterochromatin condensation (arrows). (E) Focal loss of nuclear membrane (arrows). (G) Nucleolar hole (arrows). (H) Accumulation of mitochondria around nucleus (arrows).

We performed muscle biopsy on 13 patients. In the L-CMD group, the muscle biopsies of patients 7, 9, 11, and 13 revealed inflammatory cell infiltration. Two biopsy specimens had dystrophic changes accompanied by inflammation. Two biopsy samples had inflammatory myopathy (Fig 1B), and two had mild myopathic changes (Fig 1C). The EDMD group had only dystrophic changes, observed as increased variation in fiber size and proliferation of connective tissue. We examined the muscle cell ultrastructure of 11 patients, finding focal or extended filament disruption, indistinct sarcomeres, many vacuoles in the muscle fibers, increased adipose and connective tissue, abnormal nuclear morphology with heterochromatin condensation, focal loss of nuclear membrane, accumulation of mitochondria around the nucleus, nuclear bands, and nucleolar holes (Fig 2). There was abnormal nuclear morphology in 10 patients.

### Mutation analysis

**Fig 3:**
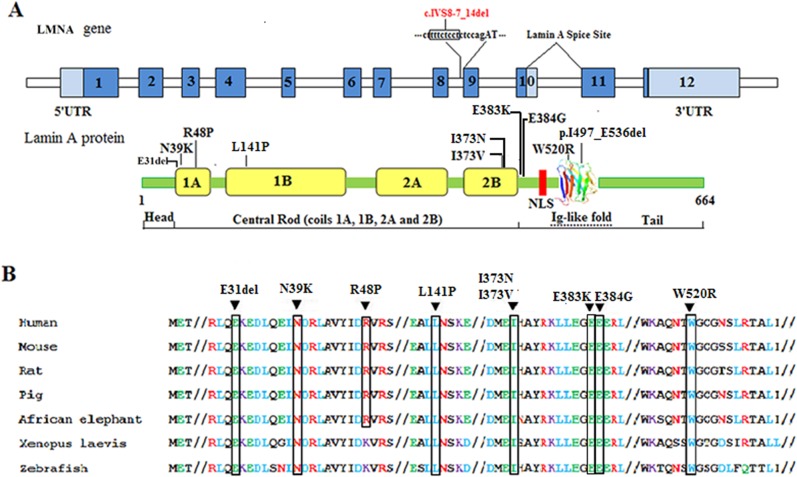
Schematic of the *LMNA* gene and lamin A protein indicating mutations. (A) Schematic of the *LMNA* gene and lamin A protein. Lamin A is encoded by exons 1–12, whereas lamin C terminates at exon 10 with six unique amino acids at its C-terminal. The alternative splice site for lamin A is indicated. Sequence variations affecting the splice donor or acceptor sites that lead to disease are shown for IVS-8. Missense mutations and deletions are indicated on the lamin A protein, where the head, central rod, and tail domain (incorporating the nuclear localization signal [NLS], and Ig-like fold) are indicated. Novel sequence variants are shown above the gene/protein. (B) Illustration of the evolutionary conservation of residues associated with novel missense mutations located in the coding region of *LMNA*. Sequences were obtained from the online database, Swiss-Prot (http://expasy.org/sprot/) and aligned using the Align online tool (http://www.uniprot.org/align/).

Direct sequencing of PCR-amplified genomic DNA detected 19 mutations in the 21 patients. The diagnosis and mutation of each patient are listed in Table 2. Nine heterozygous mutations: c.91G>A (p.E31K), c.94_96delAAG (p.K32del), c.116A>G (p.N39S), c.745C>T (p.R249W), c.746G>A (p.R249Q), c.832G>C (p.A278P), c.1124C>G (p.A375G), c.1580G>C (p.R527P), and c.1540T>A (p.W514R), have been reported previously, while the other 10 were novel mutations: c.91_93delGAG (p.E31del), c.117T>G (p.N39K), c.143G>C (p.R48P), c.422T>G (p.L141P), c.1117A>G (p.I373V), c.1151A>G (p.E384G), c.IVS8-7_14del (p.I497_E536del), c.1558T>C (p.W520R), c.1118T>A (p.I373N), and c.1147G>A (p.E383K). To evaluate the c.IVS8-7_14del mutation and confirm its pathogenicity and influence on splicing, we examined the consequences of the mutation at cDNA level in the peripheral blood leukocytes of patient 13 and his unaffected father as a control using RT-PCR and verified a heterozygous exon 9 deletion in patient 13; the deletion was not found in his father. Among the previously reported mutations, only c.1580G>C was found in the father of patient 15, who displayed the same symptoms as his daughter. The other mutations were not found in the parents, indicating that they occurred de novo. All new *LMNA* mutations were not found in the control population (as well as in the 1000 Genomes database), who did not have any known muscle disease. Novel sequence variants and an illustration of the evolutionary conservation of residues associated with novel missense mutations are shown in Fig 3.

**Table 2:** Phenotype and genotype of patients.

Patient No.	Phenotype	Mutation	*LMNA* exon/ intron	Predicted amino acid change	Protein domain	p.r.or novel	Inheritance
1	L-CMD	c.91G>A	exon1	p.E31K	Coil 1a	p.r.	/
2	L-CMD	c.94_96delAAG	exon1	p.K32del	Coil 1a	p.r.	/
3	L-CMD	c.91_93delGAG	exon1	p.E31del	Coil 1a	Novel	De novo
4	L-CMD	c.94_96delAAG	exon1	p.K32del	Coil 1a	p.r.	De novo
5	EDMD	c.116A>G	exon1	p.N39S	Coil 1a	p.r.	/
6	L-CMD	c.117T>G	exon1	p.N39K	Coil 1a	Novel	De novo
7	L-CMD	c.143G>C	exon1	p.R48P	Coil 1a	Novel	De novo
8	L-CMD	c.422T>C	exon2	p.L141P	Coil 1b	Novel	De novo
9	L-CMD	c.745C>T	exon4	p.R249W	Coil 2b	p.r.	De novo
10	EDMD	c.746G>A	exon4	p.R249Q	Coil 2b	p.r.	De novo
11	L-CMD	c.1117A>G	exon6	p.I373V	Coil 2b	Novel	De novo
12	L-CMD	c.1151A>G	exon6	p.E384G	Coil 2b	Novel	De novo
13	L-CMD	c.IVS8-7_14del	intron8	p.I497_E536del	Tail (Ig-fold)	Novel	De novo
14	EDMD	c.1558T>C	exon9	p.W520R	Tail (Ig-fold)	Novel	De novo
15	EDMD	c.1580G>C	exon9	p.R527P	Tail (Ig-fold)	p.r.	Paternal
16	EDMD	c.1124C>G	exon6	p.A375G	Coil 2b	p.r.	De novo
17	EDMD	c.832G>C	exon5	p.A278P	Coil 2b	p.r.	De novo
18	L-CMD	c.1118T>A	exon6	p.I373N	Coil 2b	Novel	De novo
19	L-CMD	c.1147G>A	exon6	p.E383K	Coil 2b	Novel	De novo
20	L-CMD	c.745C>T	exon4	p.R249W	Coil 2b	p.r.	De novo
21	EDMD	c.1540T>A	exon9	p.W514R	Tail (Ig-fold)	p.r.	De novo

p.r.: Previously reported.

### Immunofluorescence analysis and transfection of HEK 293 cells

**Fig 4:**
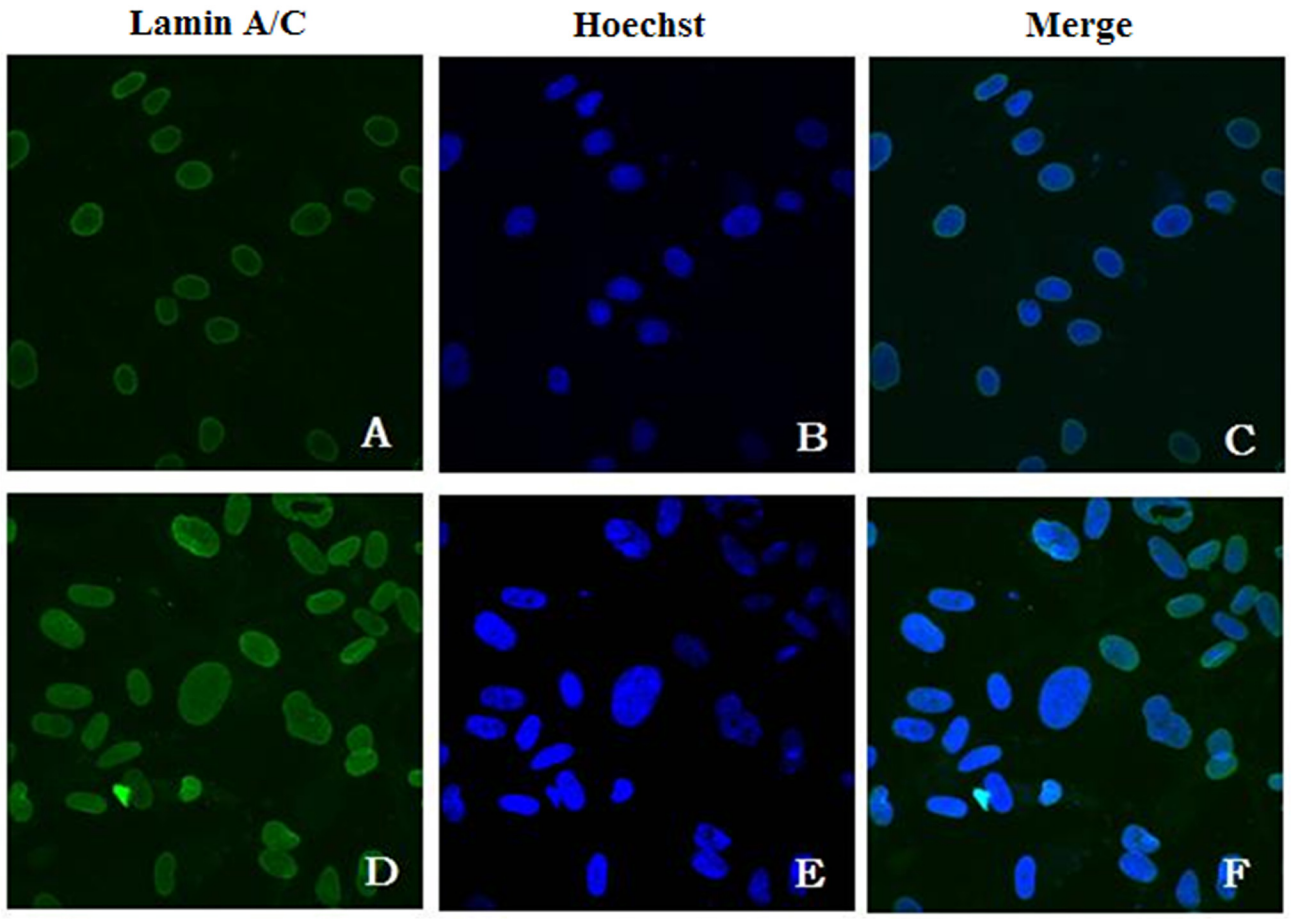
Abnormal fibroblast nuclear morphology. Immunofluorescence staining of (A–C) control and (D–F) patient 7 was carried out using antibodies against (A, D) lamin A/C. (B, E) Corresponding Hoechst staining; (C, F) merged images.

**Fig 5:**
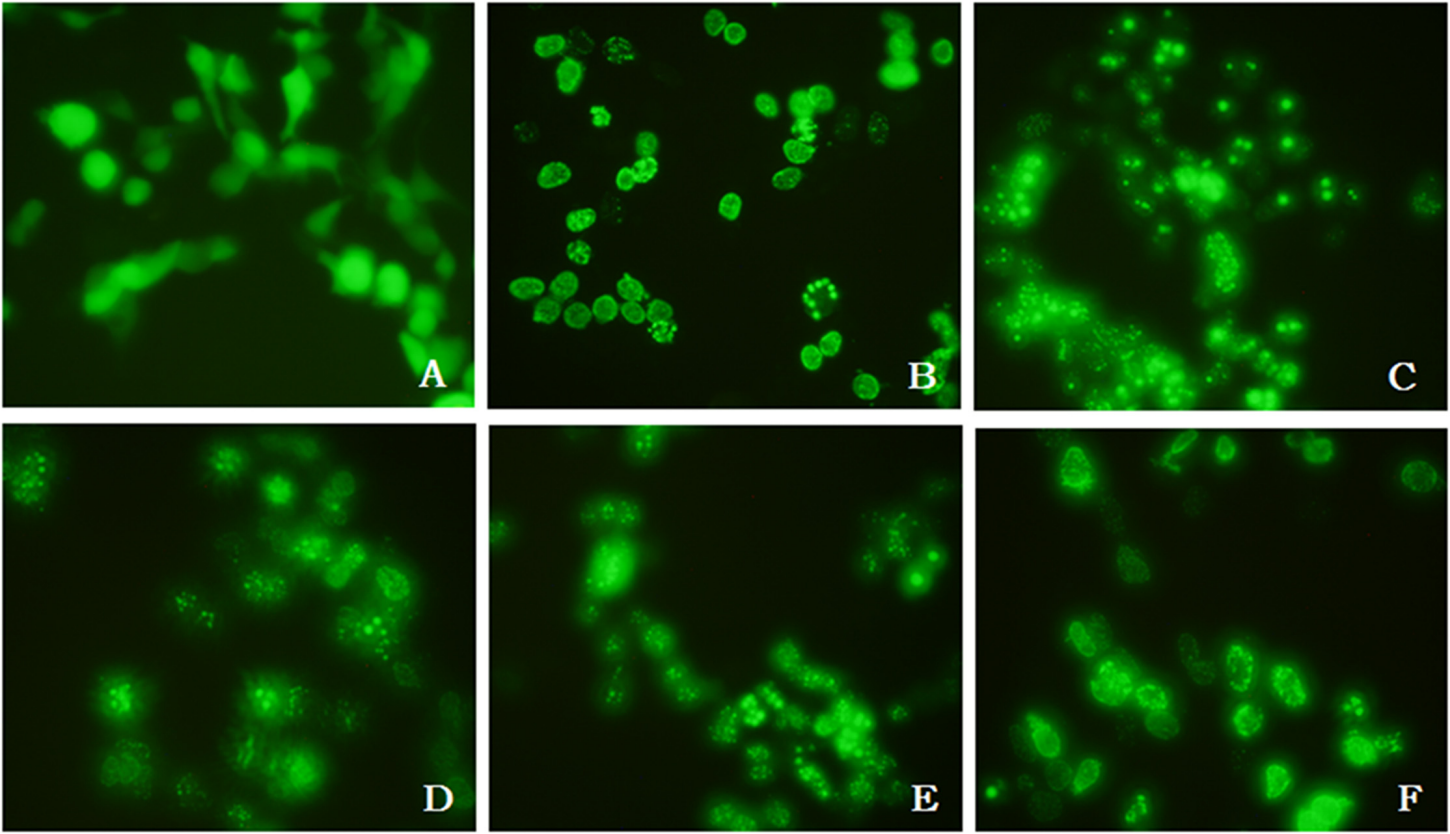
Lamin A/C mislocation in mutant-transfected HEK 293 cells. Transfection was performed using (A) GFP, (B) pEGFP-N1-LMNA, (C) pEGFP-N1-LMNA-R48P, (D) pEGFP-N1-LMNA-R249W, (E) pEGFP-N1-LMNA-I373V, or (F) pEGFP-N1-LMNA-I497_E536del. Lamin A/C of the mutants (C–F) is distributed in clusters and is mislocated compared with that of the wild type (B).

Under immunofluorescence, dermal fibroblasts from patient 7, who carries the *LMNA* ^R48P/WT^ mutation, had abnormalities (35/100 diaminophenylindole [DAPI] nuclei) such as nuclei of unequal size and abnormal morphology as compared with control skin fibroblasts (4/100 DAPI nuclei) (Fig 4). We examined the effect of the four mutations (p.R48P, p.R249W, p.I373V, p.Ile497_Glu536del) on the subcellular localization of lamin A/C after transfection of mutant constructs into HEK 293 cells. Transfection of the wild-type construct resulted in normal lamin A/C localization at the inner nuclear membrane. However, lamin A/C immunoreactivity following transfection of the mutant constructs was abnormally distributed, forming distinct aggregates (Fig 5).

## Discussion

The more recently recognized L-CMD in the Chinese population is rarely reported. However, early diagnosis is greatly important for these patients because of the high rate of morbidity and fatal arrhythmia necessitating proactive cardiac intervention [8]. A clear analysis of the associated clinical features will allow for early clinical recognition and thus genetic diagnosis. This study focused on early-onset *LMNA*-related muscular dystrophy in Chinese patients.

Due to the severity of the disease, the 14 patients in our cohort clinically diagnosed with L-CMD had exhibited motor development retardation since infancy, and 10 of them had dropped head syndrome due to pronounced weakness of the neck muscles. This was accompanied by early involvement of the paraspinal muscles. The maximum motor ability achieved by patients with L-CMD is worse than that of patients with EDMD, and the condition progresses more quickly. The patients diagnosed with EDMD exhibited typical symptoms suggestive of the disease: contracture of the elbows, Achilles tendons, and rigid spine at the early stage; slowly progressive muscle weakness and scapuloperoneal atrophy in early childhood; and early signs of cardiac involvement.

With the exception of one inherited dominant heterozygous mutation, all of the mutations identified in our study were de novo heterozygous substitutions, which also acted as dominant mutations. The dominant mutation is consistent with the results reported, but is independent of the disease type or severity. The mutations reported in patients with *LMNA*-related muscular dystrophy in general are mainly present in exons 1, 4, 6, 7, and 9 of the *LMNA* gene (http://www.dmd.nl/). The mutations of the 21 patients in our study were mainly in exons 1, 4, 6, and 9. With the exception of the exon 9 skipping mutation, the mutations were mostly missense or one–amino acid deletions resulting in changes in the coil 1A, coil 2B, and tail (Ig-fold) protein domains. These domains are related to dimerization, structural stability, chromatin binding, and the binding of other interacting proteins such as emerin, retinoblastoma protein, and lamina-associated polypeptide 2α [9]. Thus, changes to these domains are likely to significantly interfere with specific lamin A/C functions and interactions, eventually resulting in disease. Although the pathogenic mechanism of individual mutations in the *LMNA* gene for pathogenesis in special tissues that account for the side range of *LMNA*-associated disorders is unknown, some mutations appear well correlated within the same disease. Therefore, analyzing each mutation and its phenotype remains essential for determining the phenotype–genotype relationship.

Mutations such as c.91G>A (patient 1), c.94_96delAAG (patient 2 and 4), c.116A>G (patient 5), c.745C>T (patient 8), c.746G>A (patient 9), c.1580G>C (patient 14), and c.832G>C (patient 17) have been reported previously (http://www.dmd.nl/).^10^ Patient 1 and a reported case with the same mutation both had L-CMD and exhibited dropped head syndrome [10]. Patient 1 died at the age of 7 years, but the cause of death remains unknown. The two patients with c.94_96delAAG were diagnosed with L-CMD. Of six reported cases with the same mutation, three each had EDMD and L-CMD (http://www.dmd.nl/). Work on a mouse model of the c.94_96delAAG mutation reported early death resulting from metabolic defects [11]. Metabolic defects have not been reported in human patients; however, patients with this mutation should be monitored for the presence of metabolic disorders. The c.116A>G mutation has been reported in three cases of L-CMD in patients of Caucasian, Arab, and Hispanic descent (http://www.dmd.nl/). Patient 5 in our series, who carried this mutation, was diagnosed with relatively early-onset EDMD, displaying Trendelenburg gait at the age of 2 years, subsequently developing elbow contracture, deformed spine, muscle weakness, and scapuloperoneal atrophy. Due to the limited information in previous reports, we could not determine the exact difference in phenotype for patient 5; we speculate that the variable severity is perhaps related to ethnic background. Both EDMD and L-CMD have been reported in patients with c.116A>G, c.745C>T, and c.746G>A mutations, again suggesting variability of severity due to additional modifying factors. The c.1580G>C mutation has been mainly reported in EDMD (http://www.dmd.nl/). Consistent with this correlation, patient 15 and her father both carry this mutation and both have EDMD. Taken together, the phenotypes of our patients and the previously reported cases with the same mutation indicate that the six previously reported mutations are all related to some form of muscular dystrophy: c.91G>A and c.745C>T are related to L-CMD, c.94_96delAAG and c.116A>G to EDMD and L-CMD, and c.746G>A and c.1580G>C to EDMD.

Ten mutations identified in our cohort are novel. Among them, nine were detected in patients with L-CMD. As the number of patients per mutation is limited, phenotype–genotype correlation in our cohort remains tentative. However, some information may be derived from the predicted consequences of the amino acid changes. For example, c.91_93delGAG (p.Glu31del) in patient 3 with L-CMD results in Glu31 deletion, while a previously reported missense mutation in Glu31 (c.91G>A, p.Glu31Lys) resulted in L-CMD. Also, c.117T>G in patient 6 resulted in L-CMD, while other Asn39 missense mutations reported have all been associated with L-CMD. Therefore, Glu31 and Asn39 may be critical residue mutations that can result in L-CMD. Although mutations of the adjacent residue Leu140 have been reported in EDMD and Werner syndrome, no Leu141 mutations have been reported [12, 13]. It is unclear whether several phenotypes are related to Leu141 alteration. One EDMD case involving a Trp520 mutation has been reported, which is the same as that for patient 14 [14], suggesting that Trp520 mutations are related to EDMD. It is noteworthy that the mouse model with exon 9 deletion (*LMNA*∆9/Δ9) resulted in Hutchinson-Gilford progeria syndrome and telomere and chromatin defects [15]. However, patient 13, who had the exon 9 skipping mutation, only presented with muscle weakness since infancy and dropped head syndrome. At the age of 2 years, patient 13 does not exhibit any symptoms of progeria, and will need further follow-up. Thus, based on our series and reported cases, there appear to be some phenotype–genotype correlations.

In addition to the presenting symptoms and clinical examination, it may be of particular interest to also include careful consideration of the muscle biopsy findings into the clinical analysis. In our study, inflammatory changes were observed in the biopsy specimens of four patients with L-CMD who carried the c.143G>C, c.745C>T, c.1117A>G, and c.IVS8-7_14del mutations, respectively. In these patients, muscle biopsy was performed when they were aged 16 months, 18 months, 8 months, and 10 months, respectively. A previous report found that histological evidence for inflammation in *LMNA*-related muscular dystrophy is related to the age at muscle biopsy and is usually seen in biopsies from infants [16]. It was reported that steroid treatment is effective for some patients with *LMNA*-related muscular dystrophy.^16^ Thus, inflammation may play an important role at onset in the pathogenesis of *LMNA*-related muscular dystrophy [17]. The option of steroid treatment therefore requires further study. However, in the four patients, short courses of steroid treatment (<6 months) did not appear to have an ameliorating effect on the disease.

Alterations of nuclear morphology have been observed in patients with *LMNA* mutations and are believed to reflect one of the pathogenic mechanisms by influencing nuclear functions such as gene transcription [18]. In our study, deformable nuclei were observed in both muscle cells (transmission electron microscopy) and skin fibroblasts (lamin A/C immunofluorescence). In addition, lamin A/C localization in HEK 293 cells transfected with mutant constructs was abnormal, resulting in focal aggregation. These findings suggest that *LMNA* gene mutations alter lamin A/C distribution, affecting mechanical stability and disrupting nucleocytoskeletal coupling [19]. The resulting abnormal nuclear morphology and altered stiffness lead to muscle cell death and tissue degeneration [20].

In addition to the progressive motor impairment, patients are at risk for developing subsequent cardiac dysrhythmias and cardiomyopathy, respiratory failure, feeding difficulty, and orthopedic involvement including scoliosis. Cardiac involvement is common and life-threatening. A decrease in blood glutathione in patients with *LMNA* gene mutations may serve as an early marker of cardiac involvement but has not been systematically studied in early-onset forms [21]. A meta-analysis of 299 patients with *LMNA* gene mutations found that 92% of patients had cardiac dysrhythmias after the age of 30 years, and there was sudden death in 42% of patients with the neuromuscular phenotype [22]. Therefore, regular cardiac surveillance is necessary for such patients. Respiratory involvement is the second most commonly reported cause of death. In patients with L-CMD, recurrent respiratory infection and respiratory failure are common before patients are 8 years old, and some require noninvasive ventilation [5, 23]. In our study, recurrent respiratory infection before the age of 2 years in three patients. Therefore, it is important to minimize the risk of respiratory infection and respiratory therapy should be administered during intercurrent respiratory infections, and to perform careful respiratory monitoring for timely initiation of respiratory support when needed. In addition to respiratory muscle weakness, spinal deformations such as excessive thoracic lordosis and scoliosis can also contribute to respiratory failure. For that reason, spinal corrective aids should be used when required [22]. Feeding difficulty and weight maintenance also should be addressed [24].

In summary, early-onset *LMNA*-related muscular dystrophy is mostly caused by de novo mutations in the *LMNA* gene. Although there are many laminopathy phenotypes, some mutations appear to have strong phenotype–genotype correlation in *LMNA*-related muscular dystrophy and would guide further mechanistic studies of the pathogenesis of skeletal- and cardiac muscle–specific involvement in *LMNA* mutations.
